# Determination of the Functional, Nutritional, and Some Quality Properties of Kefir Produced With the Addition of Germinated Chickpeas

**DOI:** 10.1002/fsn3.70860

**Published:** 2025-08-25

**Authors:** Ayşe Nur Kahve, Ebru Bayrak

**Affiliations:** ^1^ Department of Sports and Health Aksaray University Aksaray Turkey; ^2^ Department of Nutrition and Dietetics Selcuk University Konya Turkey

**Keywords:** germination, kefir, prebiotics, principal component analysis

## Abstract

Global population growth and rising health consciousness have increased the demand for sustainable and functional foods. This study aimed to enhance the nutritional value of chickpeas (*Cicer arietinum L*.) and evaluate the functional and quality characteristics of kefir enriched with germinated chickpeas. Germination conditions were optimized, and chickpea samples were analyzed for protein, moisture, ash, and total phenolic content. Principal component analysis (PCA) identified the optimal germination parameters. Kefir samples were prepared by incorporating ungerminated chickpeas, inulin, or optimally germinated chickpeas at concentrations of 0.5%, 1%, and 2%, and stored at 4°C ± 1°C for 7 days. Physicochemical, microbiological, and sensory analyses were performed on Day 1 and 7. The highest protein (14.99%) and total phenolic content (9.05 mg GAE/g) were observed in chickpeas soaked for 12 h at 30°C and germinated for 72 h. Kefir pH values ranged from 4.01 to 4.75 (*p* < 0.05). Increasing the concentration of germinated chickpeas significantly improved the total phenolic content (*p* < 0.05), and yeast growth was detected exclusively in these samples (*p* < 0.05). However, sensory evaluation scores declined with higher chickpea concentrations. In conclusion, kefir enriched with germinated chickpeas demonstrates enhanced nutritional and microbiological properties, indicating its potential as a functional beverage. Nevertheless, further improvements are necessary to optimize its sensory characteristics for consumer acceptance.

## Graphical abstract

Due to the increasing prominence of “sustainable nutrition” as a result of climate change, germinated foods have gained significant importance. This study aims to optimize the germination of chickpeas, a staple in developing countries, and investigate their nutritional value when added to kefir, a traditional fermented product.

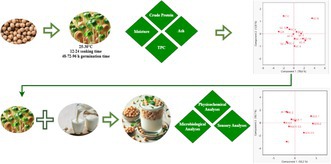

## 1 Introduction

Legumes are widely acknowledged for their crucial role in sustainable nutrition, particularly as alternative sources of plant‐based protein. However, their nutritional potential is often hindered by the presence of antinutritional compounds such as trypsin inhibitors, saponins, α‐amylase inhibitors, phytic acid, and oxalates, which reduce the bioavailability of essential nutrients (Avezum et al. 2023). Germination has emerged as a promising and cost‐effective strategy to reduce these antinutritional factors while simultaneously improving the sensory qualities and functional properties of pulse grains. This bioprocessing technique enhances the nutritional profile of both legumes and cereals, thereby contributing to food security and dietary quality (Goharpour et al. 2025). Among leguminous crops, chickpeas ( *Cicer arietinum* L.) are of particular dietary importance due to their rich composition of nutrients and bioactive compounds (Raza et al. 2019). They represent an affordable, high‐quality protein source, especially for vegetarian populations and communities in developing countries where access to animal‐derived proteins is limited. In addition to their protein content, chickpeas provide significant amounts of vitamins, minerals, and complex carbohydrates, making them a valuable component of a balanced diet (Sofi et al. 2020).

The germination of chickpeas and their incorporation into functional food formulations is increasingly supported by their diverse nutritional benefits (Saeed et al. 2023; Kaur and Prasad 2021). Numerous studies have demonstrated that germination significantly enhances the nutritional quality of chickpeas by increasing crude protein levels (Ferreira et al. 2019; Xu et al. 2019; Mao et al. 2024), enriching bioactive compounds (Butkutė et al. 2018; Aisa et al. 2019; Domínguez‐Arispuro et al. 2018), and elevating the content of specific vitamins (Jeong et al. 2019; Zhou et al. 2015). In addition, there is mounting evidence indicating that germination effectively reduces antinutritional factors such as phytates, tannins, and phytic acid (Yaver 2022; Newton and Majumder 2023; Dida Bulbula and Urga 2018; Khandelwal et al. 2010), thereby improving nutrient bioavailability and overall digestibility.

In recent years, there has been a growing interest among researchers and the food industry in developing strategies to enhance the nutritional profile of food products. Among these approaches, fermentation has remained a widely adopted technique due to its dual function: extending the shelf life of raw materials and improving both the sensory attributes and nutritional value of foods (Giuffrè and Giuffrè 2024). In the context of lactic acid fermentation, carbohydrates are metabolized by lactic acid bacteria (LAB). Homofermentative LAB strains primarily produce lactic acid, while heterofermentative, Gram‐positive strains generate additional metabolites such as acetic acid, ethanol, and carbon dioxide alongside lactic acid. These facultative anaerobic, rod‐shaped microorganisms play a key role in the production of traditional fermented foods—such as yogurt, kefir, sauerkraut, and kimchi—which are widely recognized for their potential health‐promoting effects (Giuffrè and Giuffrè 2024; Javaid et al. 2025).

Fermented dairy products, in particular, are recognized as functional foods that contribute significantly to human health (Salehi 2021). Among them, kefir stands out as one of the most popular and extensively consumed fermented dairy beverages (Manab et al. 2017). Originating from the Caucasus, the Balkans, and Eastern Europe, kefir continues to be favored in many parts of the world today (Mendes et al. 2021). Traditionally, kefir is produced by inoculating pasteurized milk with kefir grains or a defined starter culture, followed by fermentation at approximately 25°C for 16 to 22 h until the pH drops to around 4.6. This fermentation process results in the formation of key metabolites such as lactic acid, ethanol, and acetic acid (Güzel‐Seydim and Kök‐Taş 2019).

Functional foods are natural or processed food products that not only supply essential nutrients required for human health but also confer additional health benefits, such as enhancing immune function and reducing the risk of disease (Granato et al. 2020). Within this category, nutraceuticals represent a particularly prominent group (Damián et al. 2022). Zeisel (1999) suggested that prebiotics may also be classified as nutraceuticals, particularly when incorporated into a food matrix rather than consumed in supplement form. Today, functional foods encompass a wide array of products, with milk and dairy‐based items constituting a substantial share of the market. In this context, increasing the nutraceutical potential of kefir has garnered considerable attention, encouraging the incorporation of targeted ingredients that offer distinct functional and health‐promoting properties (Aiello et al. 2020). Consequently, numerous studies have explored the addition of prebiotics to dairy products using various strategies (Balthazar et al. 2018; Shafi et al. 2019; Souza et al. 2019). Prebiotics such as inulin, wheat bran, psyllium, oyster mushroom extract, and gum arabic have been widely investigated for their functional benefits (Vital et al. 2015; El‐Shenawy et al. 2016). As a result, efforts to enhance the health‐promoting properties of fermented products—especially fermented milk—through prebiotic enrichment have come to the forefront. In addition to delivering nutrients and bioactive compounds, fortifying functional beverages with prebiotics can stimulate the growth of beneficial microorganisms during fermentation. Notably, chickpeas are a rich source of oligosaccharides, which are increasingly recognized for their prebiotic potential (Pandae et al. 2024). Both in vivo and in vitro studies have demonstrated that dietary fibers, due to their prebiotic properties, support the viability of probiotic strains such as *Bifidobacterium lactis* Bb‐12 and *Lactobacillus acidophilus* La‐5. In addition, supplementation with chickpea flour has been reported to promote the growth of *Lactobacillus delbrueckii* ssp. *bulgaricus* in yogurt (Gibson et al. 2017; Martinez‐Villaluenga et al. 2006). Yogurt enriched with aqueous chickpea extract was found to contain higher counts of *Streptococcus thermophilus* compared to the control without the extract (Zare et al. 2012). This finding suggests that the high dietary fiber and protein content of chickpeas may serve as a beneficial component for enhancing the microbiological quality and nutritional value of fermented dairy products (Abd Rabo et al. 2019).

One of the aims of this study is to enhance the nutritional quality of chickpeas, which have high production potential in Turkey, and to facilitate their integration into the food industry while making them more accessible to consumers. In this context, improving the nutritional value of chickpeas through germination and utilizing them as an alternative high‐quality protein source represents a key objective of the research. The main objective of this study was to determine the functional properties, nutritional value, and some quality characteristics (antioxidant activity, total phenolic matter, etc.) of kefir produced with the addition of germinated chickpeas.

## 2 Materials and Methods

### 2.1 Chemicals and Reagents

Sulfuric Acid, Boric Acid, Sodium Hydroxide, Folin Reagent, Sodium Hypochlorite, Methanol, YGC, MRS, and PCA medium were obtained from Merck (Darmstadt, Germany). Ethyl Alcohol and Sodium Carbonate were obtained from Tekkim (Bursa, Turkey), DPPH (St. Louis, Missouri, USA).

### 2.2 Materials

In this study, the chickpea type named Çiftçi, which is registered by the Directorate of Eskişehir Geçit Kuşağı Agricultural Research Institute, was used for the optimization of chickpea. According to the results of the analysis provided by the directorate of the institute, it was reported that the related species was registered in 2021, its average size was 9 mm, and 100 grain weight varied between 35.9 and 48.5 g. Chickpeas, originating from Turkey, were harvested in June 2023. Harvested seeds were stored in dry and cool conditions (below 20°C, relative humidity < 60%) until analysis. At the time of the experiments, the seeds were less than a year old, providing an advantage in terms of quality in germination and biochemical analyses. Lyophilized powder dry kefir culture was used in the study. For the production of kefir, 3.1% fat UHT cow's milk was used to ensure that the lyophilized powdered dry kefir culture complies with the instructions for use, accessibility, and standard content.

### 2.3 Procedure of Germination

The germination procedure was carried out by making minor modifications to the method proposed by Yılmaz Tuncel et al. (2025). In the preliminary trials of the research, three factors were taken as the basis for the germination of chickpea: germination time, germination temperature, and soaking time in water. In order to determine the groups of chickpeas, it was taken into consideration that mold did not occur in chickpeas depending on the germination temperature and germination time, and that no undesirable color was formed in the smell, color, and texture of chickpeas. Accordingly, the germination temperature of chickpeas was determined as 25°C–30°C, soaking time as 12–24 h and germination time as 48–72–96 h.

**FIGURE 1:**
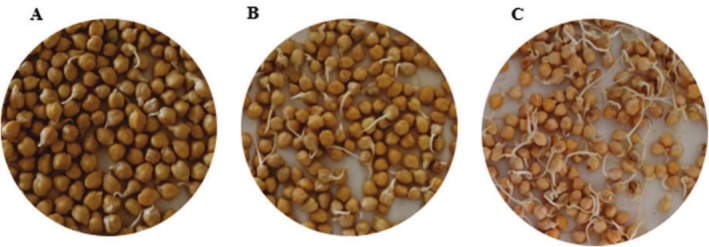
Germination of chickpea sample for 2 days (48 h, A), 3 days (72 h, B), 4 days (96 h, C).

In the preliminary experiment where chickpeas were germinated and in the optimization phase determined, the samples were weighed as 300 g. Then 300 mL of a solution containing 1% sodium hypochlorite (NaHCl) was added and kept for 45 min. After the waiting phase, chickpeas were washed with tap water for 30 min to remove NaHCl. Before starting the germination process, 1000 mL of pure water was added to the chickpeas and soaked for 12 and 24 h. After this process, the experimental design was formed by germinating the chickpeas between cotton cloth in the dark environment in the air conditioning cabinet (Nüve, TK 252, Turkey) at 25°C–30°C temperatures and 85% ± 5% relative humidity for 48, 72, and 96 h. Germination of chickpeas is shown in Figure 1. During germination, pure water was sprayed on the chickpeas covered with cotton cloth every 12 h to keep the chickpeas moist. After the germinated chickpea samples were allowed to dry in an oven at 40°C for 5 h, the samples were pulverized with a laboratory‐type blender, sieved, and stored at −18°C for analysis.

### 2.4 Analyzes Applied to Germinated Chickpea

#### 2.4.1 Determination of Crude Protein

Crude protein was determined by the Kjeldahl method according to AOAC (Horwitz 1975).

#### 2.4.2 Determination of Moisture Content

The moisture content of chickpeas was determined from the weight loss following the evaporation process using the AACC (2000) method. Weighed 1.5 g of each chickpea sample into tared glass petri dishes. The samples were spread homogeneously over the whole dish and dried in an oven (Nüve, MF 106, Turkey) (135°C, 120 min). It was then allowed to cool in a desiccator (with granular glica gel) for 30 min.

#### 2.4.3 Determination of Ash Content

The ash content of chickpeas was determined by burning in a muffle furnace (Nüve, MF 106, Turkey) at 550°C until constant weight (AACC 2000).

#### 2.4.4 Extraction of Phenolic Compounds

3 g of all samples were taken and shaken with 15 mL acidified methanol (1:80:10, v/v, HCl/Methanol/water) for 2 h at room temperature. The extracts were then centrifuged with a macrocentrifuge (Nüve, NF 3000R, Turkey) at 3000 rpm at 4°C for 10 min, and the supernatant was obtained.

#### 2.4.5 Total Phenolic Content (TPC)

Total phenolic matter was determined according to the method described by Fernandez‐Orozco et al. (2006). The supernatant (0.1 mL) was mixed with Folin–Ciocalteu reagent (0.5 mL, 10% v/v, in water) and sodium carbonate solution (1.5 mL, 20% v/v, in water) and incubated for 2 h at room temperature. Absorbance was measured at 760 nm using a spectrophotometer (Hitachi, U‐1800, Japan). The total phenolic content of the samples was calculated as gallic acid equivalent (mg GAE kg^−1^), taking into account the moisture content (%) based on the following formula.

$$\mathrm{TPC}\;\left(\mathrm{mg}\;\mathrm{GAE}/\mathrm{kg}\;\mathrm{dry}\;\text{matter}\right)=\frac{\mathrm{TPC}\;\left(\mathrm{mg}\;\mathrm{GAE}/\mathrm{kg}\;\text{fresh matter}\right)}{1-\frac{\text{moisture}\;\left(\%\right)}{100}}$$

### 2.5 Enriching Kefir With Germinated Chickpeas

#### 2.5.1 Preparation of Kefirs and Formation of Groups

**FIGURE 2:**
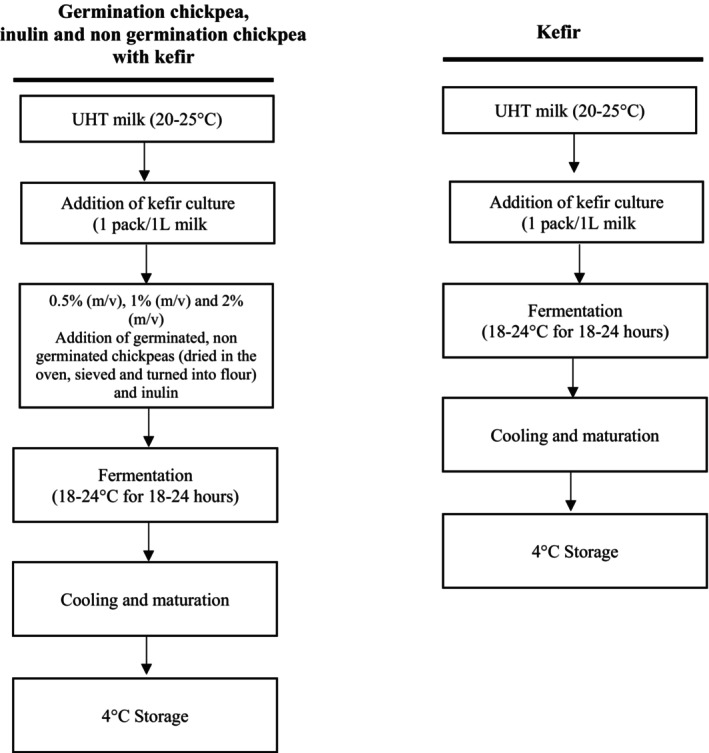
Production flowchart of kefir.

The optimization phase of the germinated chickpeas to be used in kefir production was completed, and the group to be added was determined. The ratios to be used in the enrichment of kefir were determined as 1% (m/v), 2% (m/v), and 3% (m/v) in preliminary trials, but when evaluated in terms of consistency, odor, and taste, the laboratory team decided on 0.5% (m/v), 1% (m/v), and 2% (m/v). The storage period of kefir was determined as the 1st, 7th, and 14th days, and sensory analysis could not be performed due to the negative taste and odor changes that occurred in kefir on the 14th day during the preliminary trials. For this reason, it was decided to determine the 1st and 7th days as the storage period in the method. The production flow chart of kefirs is given in Figure 2.

Kefir culture (1 pack/1 L milk) was added to the room temperature UHT cow milk used in kefir production, and kefirs were left for fermentation. Kefir with germinated chickpea (GCK), kefir with inulin (IK) and kefir with ungerminated chickpea (NGCK) groups fermented at 0.5% (m/v), 1% (m/v), and 2% (m/v), respectively. The kefirs that completed the fermentation process were kept in the refrigerator at +4°C for 1 day and matured. Physicochemical, physical, and microbiological analyses of the matured and ready‐to‐consume kefirs were carried out.

### 2.6 Analyzes Applied to Kefir

#### 2.6.1 Serum Separation

To determine the serum separation of kefir samples, empty centrifuge tubes were first weighed. Then, 25 g kefir sample was placed in a centrifuge tube and centrifuged (Nüve, NF 3000R, Turkey) at 1250 g for 10 min at 4°C. The supernatant portion of the centrifuged tubes was poured out and the centrifuge tubes containing pellets were weighed (final weighing). The values of the weighing results were calculated by placing them into the equation given below (Şahin et al. 2025).

$$\begin{array}{cc} \text{Serum Separation}\;\left(\%\right)\;=\; & \left(\text{Final Weighing}-\text{Weight of the centrifuge tube}\right)\\ & /\left(\;\text{Sample Amount}\right)\times 100 \end{array}$$

#### 2.6.2 Determination of pH

The pH of all samples was determined directly on Day 1 and 7 using a digital pH‐meter with a combined electrode (Hanna Instruments, HI2211, USA).

#### 2.6.3 Determination of Total Titratable Acidity

10 mL of kefir sample to be analyzed was taken and 10 mL of distilled water was added. To the diluted and homogenized kefir sample, 0.5 mL of 1% phenolphthalein indicator was added and titrated with 0.1 N NaOH for 30 s until a permanent light pink color was formed. Titratable acidity was calculated in terms of lactic acid as g/100 g using the following formula (Metin and Öztürk 2016).

$$\text{Titratable Acidity}\;\left(\%\right)=\left[V\times F\times \mathrm{mEq}\times 100\right]/M$$

V: Volume of 0.1 N NaOH consumed (mL)

F: NaOH factor (If the exact normality is 0.1 N, this value is taken as 1)

mEq: Amount of acid equivalent to 1 mL of 0.1 N NaOH (g) (0.009008 as lactic acid)

M: Volume of the titrated sample (mL).

#### 2.6.4 Total Soluble Solid (TSS)

Total soluble solid (brix) determination of kefir samples was carried out with a handheld digital refractometer (ISOLAB, 0%–90% Brix, Germany). The refractometer was first calibrated with distilled water and then the sample was dripped directly onto the refractometer prism, and the results were recorded as % brix.

#### 2.6.5 Total Phenolic Content (TPC)

For the determination of total phenolic content, the method of Škerget et al. (2005) was followed. Folin–Ciocalteu solution was prepared according to the protocol in the work of Hussein et al. (2020). 2 mL of the samples were taken, 5 mL of 80% methanol was added, mixed with vortex for 30 s, and kept in a water bath at 25°C for 2 h. It was then centrifuged at 4000 rpm for 10 min at 4°C. The filtered supernatants were used for both total phenolic and antioxidant activity determinations. 500 μL of the supernatants were taken, 2.5 mL of 10‐fold diluted Folin–Ciocalteu solution was added, and waited for 2 min. Following this, 2 mL of 7.5% Na_2_CO_3_ (Merck, Germany) solution was added to the mixture and incubated for 30 min in the dark. The results of the samples were then read with a spectrophotometer at 760 nm. From the absorbance readings, the total phenolic content of the samples was calculated to be equivalent to gallic acid (mg GAE kg^−1^).

#### 2.6.6 DPPH Radical Scavenging Activity

For antioxidant activity, the colorimetric method used by Von Gadow et al. (1997) based on the inhibition of 2,2‐diphenyl‐1‐picrylhydrazyl (DPPH) radical was used with some modifications. For the measurement of antioxidant activity, 100 μL of sterilized samples were mixed with 6 × 10^−5^ M DPPH (Sigma Aldrich, USA) solution prepared in 4 mL of methanol and kept at room temperature in the dark for 30 min. The absorbance of the solutions was read against water at a wavelength of 516 nm. The DPPH radical scavenging activity was calculated using the following formula:

$$\text{DPPH scavenging activity}\;\left(\%\right)=\left(\frac{A0-A1}{A0}\right)\times 100$$

A0 = absorbance of the control

A1 = absorbance of the sample

#### 2.6.7 Determination of Calcium and Phosphor Content

The supernatants of the kefir groups were filtered before analysis. These supernatants were then digested in a closed microwave system (Cem‐MARS Xpress, USA) using 5 mL 65% HNO_3_ and 2 mL 35% H_2_O_2_. The volumes of the incinerated samples were completed to 20 mL with ultra deionized water, and the mineral contents were determined by ICP OES (EXPEC 6500) (Ozcan 2010).

#### 2.6.8 Microbiological Analysis

Under aseptic conditions, 10 to 90 mL of sample was added to 90 mL of physiological saline (0.85%) and diluted 1:9. Standard serial dilutions from 10^−1^ to 10^−8^ were prepared from the samples and made ready for cultivation. After serial dilutions, inoculation was made on appropriate media using the smear plate method, and viable counts were made at the end of appropriate incubation (Şahin et al. 2025).

##### 2.6.8.1 Lactic Acid Bacteria Count

Man Rogasa Sharp (MRS) agar (Merck/Germany) medium was used for *Lactobacillus* spp. count. After inoculation, Petri dishes were incubated at 30°C for 72 h (ISO 1998). The colonies observed in the petri dishes after the incubation period were counted.

##### 2.6.8.2 Total Mesophilic Aerobic Bacteria Count

Total mesophilic aerobic bacteria count (TMAB) was performed according to the method of ISO (1998). For enumeration, Plate Count Agar (PCA) (Merck, Germany) medium was inoculated using the smear culture method and left to incubate under aerobic conditions at 30°C for 2 days.

##### 2.6.8.3 Yeast Count

Yeast Extract Glucose Chloramphenicol agar (YGC, Merck, Germany) medium was used for yeast count. The medium was sterilized at 121°C for 15 min and inoculated by smear culture method after solidification. Petri dishes were incubated under aerobic conditions at 25°C for 5 days and prepared for counting (Harrigan 1998).

#### 2.6.9 Sensory Analysis

Kefir samples were evaluated by 10 panelists aged 26–40, consisting of faculty and staff experienced in sensory analysis. Panelists were selected who were familiar with the properties of kefir and frequently participated in sensory analysis (Lawless and Heymann 1999; Ertekin and Guzel‐Seydim 2010). The appearance, odor, taste, and texture criteria determined by preliminary trials were arranged according to the form created by Kök‐Taş (2010).

### 2.7 Statistical Analysis

The study was carried out in two replicates and three parallels, and statistical analyses were performed using the package program 22.0.0 (SPSS Inc., Chicago, USA). First, the normality distribution of the data was tested, and it was determined that the data showed a normal distribution according to the results of the Shapiro–Wilk test. Then, one‐way ANOVA, a parametric test, and Duncan's multiple comparison test were used for statistical evaluation of normally distributed data, and the differences of kefir samples both at different storage days and between groups were tested at a 5% confidence interval (*p* < 0.05).

In the study, Principal Component Analysis (PCA), a statistical method, was used to determine the most successful germinated chickpea group and the most multifunctionally superior kefir group. PCA analyses were performed using the JMP Pro 16.0.0 program, and the data were analyzed directly without using any conversion tool. Before starting the principal component analysis, Bartlett's test of sphericity was applied to test the suitability of the data for this analysis. According to Demirhan and Bozkurt (2010), if the calculated *p* value is less than the significance level, it is accepted that the data obtained are statistically suitable for PCA. In the Bartlett test, it was determined that the *p* value was 0.0001 and lower than the alpha value. Thus, the PCA suitability of the data was confirmed. In the selection of principal components or factors, the Kaiser criterion was observed, and only factors with Eigenvalues > 1 were used.

## 3 Results and Discussion

### 3.1 Crude Protein, Moisture, Ash, and Total Phenolic Contents of Chickpeas

The values of chickpeas germinated under various conditions and control group chickpeas are given in Table 1.

**TABLE 1:** Chemical properties of chickpea groups.

Chickpea groups	Soaking time (h)	Temperature (°)	Germination time (h)	Crude protein (%)	Moisture (%)	Ash (%)	Total phenolic content (TPC) (mg GAE/g)
GC‐1	12	25	48	13.36 **±** 0.57^ab^	64.14 ± 3.45^ab^	1.21 ± 0.12^c^	5.94 ± 0.01^d^
GC‐2	12	25	72	12.59 **±** 0.24^b^	55.77 ± 4.11^bc^	1.14 ± 0.09^d^	4.54 ± 0.04^g^
GC‐3	12	25	96	13.86 **±** 0.29^ab^	63.43 ± 6.24^ab^	1.48 ± 0.15^b^	6.58 ± 0.01^b^
GC‐4	24	25	48	12.54 **±** 0.74^b^	55.80 ± 5.22^bc^	1.14 ± 0.11^d^	5.93 ± 0.01^d^
GC‐5	24	25	72	12.94 **±** 0.66^b^	59.89 ± 4.56^b^	1.27 ± 0.13^c^	5.75 ± 0.01^e^
GC‐6	24	25	96	13.39 **±** 0.70^ab^	59.58 ± 5.01^b^	1.45 ± 0.24^b^	4.99 ± 0.02^f^
GC‐7	12	30	48	13.10 **±** 0.19^ab^	56.29 ± 4.76^bc^	1.08 ± 0.05^de^	4.48 ± 0.04^g^
GC‐8	12	30	72	14.99 **±** 0.72^a^	66.81 ± 6.89^a^	1.64 ± 0.17^ab^	9.05 ± 0.06^a^
GC‐9	12	30	96	14.25 **±** 0.46^a^	63.82 ± 5.98^ab^	1.80 ± 0.08^a^	6.66 ± 0.01^b^
GC‐10	24	30	48	14.58 **±** 0.43^a^	62.45 ± 6.76^ab^	1.56 ± 0.12^b^	6.52 ± 0.02^b^
GC‐11	24	30	72	14.02 **±** 0.25^ab^	57.64 ± 3.45^bc^	1.25 ± 0.05^c^	5.70 ± 0.04^e^
GC‐12	24	30	96	12.27 **±** 0.31^b^	52.90 ± 4.45^c^	1.13 ± 0.09^d^	6.05 ± 0.03^c^
C_12_	12			11.40 **±** 0.54^c^	57.62 ± 5.01^bc^	0.70 ± 0.02^f^	5.94 ± 0.04^d^
C_24_	24			11.44 **±** 0.45^c^	48.12 ± 4.23^d^	0.94 ± 0.09^e^	4.93 ± 0.05^f^

*Note:* Different lowercase letters in the same column indicate a significant difference between groups. (*p* < 0.05).

Abbreviations: C_12_, control chickpeas soaked for 12 h; C_24_, control chickpeas soaked for 24 h; GC, Germination Chickpea.

According to Table 1, the protein percentages of ungerminated chickpeas in C_12_ and C_24_ were 11.40% and 11.44%, respectively, and the highest protein percentage value was found in the GC‐8 group with 14.99%. When the protein value of this group was compared with the control group, it was determined that an increase of approximately 24% was achieved. There are many studies in the literature showing that germination increases the protein value of legumes (AlJuhaimi et al. 2024; Winarsi et al. 2020; Aktas et al. 2022). In a study, the protein content of ungerminated chickpeas was 20.22% ± 0.15%, while the protein content of optimally germinated chickpeas was 23.54% ± 0.21% and an increase was observed (Domínguez‐Arispuro et al. 2018). Several biochemical mechanisms contribute to the increase in protein content observed during the germination process. The first is the enhancement of proteolytic enzyme activity, which leads to the breakdown of storage proteins into small peptides and free amino acids, thereby increasing the measurable nitrogen content (Diniz et al. 2025). Another mechanism is the reduction of antinutritional factors such as phytates, tannins, and trypsin inhibitors during germination, which enhances the bioavailability of proteins—that is, their usability within the body (Johnson et al. 2020). In addition, the potential synthesis of new proteins during embryonic development may also contribute to the observed increase in total protein content (Bagarinao et al. 2024).

In a study investigating bread production with germinated chickpea flour, the protein value of chickpea flour was 187.83 ± 1.44 g/kg^−1^, while the protein value of germinated chickpea flour was 206.34 ± 1.14 g/kg^−1^ (Domínguez‐Arispuro et al. 2018). However, in another study, while the protein value of ungerminated chickpea was 16.73 ± 2.14, this value decreased to 15.39 ± 0.54 in chickpea subjected to 24 h of germination (Dida Bulbula and Urga 2018). When the results of this study were compared with the studies in the literature, it was found that the protein value of Çiftçi chickpea type was low. This situation revealed that the purpose of using low value‐added chickpea type in the study overlapped. However, similar to the studies in the literature, it was determined that the protein values increased in all groups with the germination process applied.

When the germinated chickpea groups were analyzed, the percentage of GC‐8 was found to be 66.81% and it had the highest moisture value. Among the chickpea groups, the moisture content of GC‐8 increased by 15.7% with germination (Table 1). In a study on the germination of Desi and Kabuli chickpeas, it was observed that the moisture content increased as the germination time increased. The ungerminated moisture contents of Desi and Kabuli chickpeas were found to be 10%, 30% for desi chickpea variety, and 40% for kabuli chickpea variety after 48 h of germination. After 96 h of germination, the moisture content of two chickpea varieties increased to 50% (Khalil et al. 2007). In a study conducted by Sofi et al. (2020), 3.72% dry matter loss was observed in germinated chickpea between 0 and 48 h. The loss of chickpea grain dry matter during germination may be due to leaching of substances from the grains and activation of the metabolic process (Chavan et al. 1987). Similar findings on dry matter loss by germination of finger millet have been reported in another study (Goharpour et al. 2025; Mbithi‐Mwikya et al. 2000).

When the ash content of chickpeas was analyzed, it was found that the GC‐8 group had the highest ash value (Table 1). The germination process increased the ash content of the GC‐8 group by 134.2%. There are many studies indicating that germination increases ash content (Polat et al. 2020; Kılınçer and Demir 2019; Kumar et al. 2020). In a study, the nutritional quality of legumes was examined by germination, and it was observed that two legume samples (green lentils decreased and mung beans increased) had different effects on ash content (Şenlik and Alkan 2023). It is reported that the increased ash content with the germination process is the result of the decrease in bound minerals due to the decrease in phytate. In addition, the increase in ash content can be attributed to the increase in certain minerals such as calcium, zinc, and iron (Chinma et al. 2022).

According to these results, the GC‐8 group gave the highest total phenolic matter value with a value of 9.05 mg GAE/g at 30°C and 72 h (Table 1). It was found that the TPC value of the GC‐8 group increased by 52.3% with germination. Enzymes activated during germination can destroy the cell walls around the plant, which can lead to the release of phenolic compounds and a subsequent increase in total phenolics (Wu et al. 2013). Many studies have shown that seed germination can increase the phenolic compound content (Zhang et al. 2015; Zhou et al. 2015; Xu et al. 2009). While López‐Amorós et al. (2006) and Xu et al. (2018) reported that the levels of phenolic compounds reduced during the germination of lentils, other researchers found that the levels of phenolic compounds increased during the germination of lentils (Shahidi and Yeo 2016). During seed germination, three key processes are involved in the metabolism of phenolic compounds. Initially, the synthesis of natural phenolic compounds begins with aromatic amino acids or glucose, a process that can occur as seeds germinate. Aromatic amino acids, such as phenylalanine, are produced and converted into phenolic acids in the cytosol through pathways like oxidative pentose phosphate, glycolysis, and the shikimate pathway (Herrmann and Weaver 1999). Phenolic acids are subsequently transformed into stilbenes, flavonoids, and coumarins within the endoplasmic reticulum. These phenolic compounds may then undergo polymerization or form complexes with macromolecules like polysaccharides, proteins, and lipids, which can be stored in the cell walls or vacuoles (Shahidi and Yeo 2016). Secondly, enzymes break down macromolecular nutrients, leading to the release of phenolic compounds from their bound states (Paucar‐Menacho et al. 2017). Third, phenolic compounds are utilized to neutralize free radicals or serve as intermediates in signaling pathways. The type of seed and the germination conditions are key factors influencing the transformation of phenolic compounds in these three stages during germination. As a result, it is expected that the total phenolic content and antioxidant activity may either increase or decrease following a complex germination process (Xu et al. 2020). There are many studies reporting that germination increases the total amount of phenolic substances (Mitharwal and Chauhan 2022; Demirhan and Bozkurt 2010; AlJuhaimi et al. 2024; Dziki et al. 2015).

**FIGURE 3:**
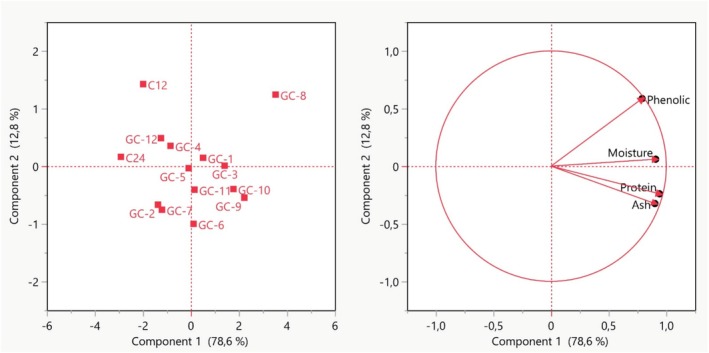
Evaluation of chemical analyses of chickpeas by PCA.

Figure 3 shows the projections of the variables used in the study against the 2 factors with the highest variance. After PCA analysis, it was determined that the eigenvalues of the 2 main components explained 91.39% of the total variance. The variances of these two factors were calculated as 78.64% and 12.75%. When the PCA analysis results of the chickpeas subjected to the germination process were examined, it was found that all groups except the GC‐8 group were clustered in the middle of the plane. It is seen that the GC‐8 group is separated from the other chickpea groups and clustered in the upper right corner of the plane. When the data are checked, it is evident that this significant difference is mainly due to the increase in total phenolic compounds and the accompanying high protein content. It was determined that the K12 group, which was separated from the middle group and located in the upper left corner of the plane, was separated from the main group because it had the lowest ash value. Looking at the projection of the analyses in Figure 3, it was determined that the analysis that had the most positive effect on the principal component analysis was the TPC.

### 3.2 Results Associated With Kefir Enriched With Germinated Chickpeas

Changes in some chemical properties of C, NGCK, IK, and GCK were examined on the 1st and 7th days of storage at 4°C ± 1°C, and the statistical analysis results of the changes in chemical properties were evaluated.

According to the results in Table 2, the highest serum separation was observed in the NGCK‐0.5 group, while the lowest serum separation was observed in the IK‐2 group. On the 7th day of storage, the highest serum separation was observed in the GCK‐0.5 group and the difference was significant (*p* < 0.05). Serum separation is an important indicator to understand how stable the product is and indicates an inhomogeneous structure or a high degree of gel instability in fermented milk products (Rasika et al. 2021). The addition of inulin was found to significantly reduce serum separation (Hut and Ayar 2013). In a study investigating kefir to which whey was added, serum separation values were examined and serum separation values increased with increasing storage days (İnce‐Coşkun and Özdestan‐Ocak 2022) and were found to be compatible with our study.

**TABLE 2:** Physicochemical properties of kefir groups.

Serum separation (X̄±SD)			pH		Total titratable acidity (TTA)		Total soluble solids (TSS)	
Samples	Day 1	Day 7	Day 1	Day 7	Day 1	Day 7	Day 1	Day 7
C	32.88 ± 1.99^d‐B^	51.89 ± 1.41^c‐A^	4.54 ± 0.01^c‐A^	4.45 ± 0.1^d‐B^	0.69 ± 0.34^d‐B^	0.78 ± 0.10^c‐A^	6.5 ± 0.20^c‐A^	6.6 ± 0.17^cd‐A^
NGCK‐0.5	42.75 ± 1.53^a‐B^	52.83 ± 2.20^c‐A^	4.25 ± 0.01^e‐B^	4.28 ± 0.1^g‐A^	0.85 ± 0.10^b‐A^	0.82 ± 0.26^b‐A^	6.5 ± 0.40^c‐A^	6.4 ± 0.36^d‐A^
NGCK‐1	38.17 ± 2.17^b‐B^	48.20 ± 1.02^d‐A^	4.40 ± 0.01^d‐A^	4.39 ± 0.1^f‐A^	0.78 ± 0.17^c‐B^	0.83 ± 0.10^ab‐A^	6.8 ± 0.10^c‐A^	6.8 ± 0.17^c‐A^
NGCK‐2	38.77 ± 0.75^b‐B^	52.66 ± 0.65^c‐A^	4.39 ± 0.02^d‐A^	4.42 ± 0.1^e‐A^	0.76 ± 0.10^c‐A^	0.72 ± 0.17^d‐B^	7.2 ± 0.17^b‐A^	7.3 ± 0.10^b‐A^
IK‐0.5	26.38 ± 1.52^f‐B^	47.52 ± 1.45^d‐A^	4.01 ± 0.01^g‐B^	4.03 ± 0.1^j‐A^	0.89 ± 0.26^a‐A^	0.84 ± 0.10^ab‐A^	7.2 ± 0.20^b‐A^	7.3 ± 0.20^b‐A^
IK‐1	29.05 ± 0.22^e‐B^	47.56 ± 0.52^d‐A^	4.11 ± 0.01^f‐A^	4.13 ± 0.1^h‐A^	0.84 ± 0.17^b‐A^	0.85 ± 0.17^a‐A^	7.5 ± 0.10^ab‐A^	7.5 ± 0.26^ab‐A^
IK‐2	22.83 ± 0.97^g‐B^	49.21 ± 1.41^d‐A^	4.09 ± 0.02^f‐A^	4.08 ± 0.1^ı‐A^	0.87 ± 0.20^ab‐A^	0.83 ± 0.00^ab‐A^	7.7 ± 0.17^a‐A^	7.8 ± 0.17^a‐A^
GCK‐0.5	39.01 ± 0.89^b‐B^	67.88 ± 0.77^a‐A^	4.69 ± 0.01^b‐A^	4.65 ± 0.2^a‐B^	0.63 ± 0.10^e‐A^	0.65 ± 0.10^e‐A^	7.3 ± 0.26^ab‐A^	7.3 ± 0.17^b‐A^
GCK‐1	35.58 ± 0.90^c‐B^	65.85 ± 0.77^ab‐A^	4.75 ± 0.02^a‐A^	4.58 ± 0.1^c‐B^	0.58 ± 0.11^f‐A^	0.60 ± 0.17^f‐A^	7.4 ± 0.26^ab‐A^	7.4 ± 0.17^b‐A^
GCK‐2	34.83 ± 1.03^cd‐B^	64.66 ± 0.88^b‐A^	4.73 ± 0.02^a‐A^	4.62 ± 0.1^b‐B^	0.52 ± 0.10^g‐A^	0.55 ± 0.10^g‐A^	7.5 ± 0.10^ab‐A^	7.6 ± 0.17^ab‐A^

*Note:* Different lowercase letters in the same column indicate a significant difference between groups. (*p* < 0.05). Different capital letters on the same line indicate a significant difference between the groups (*p* < 0.05).

Abbreviations: C, Control kefir; GCK, Germinated chickpea kefir; IK, Inulin kefir; NGCK, Non germinated chickpea kefir.

When the first day pH values of the kefir groups were analyzed, the highest pH value was observed in the GCK‐1 and GCK‐2 groups, and the lowest pH value was found in the IK‐0.5 group, and there was a significant difference (*p* < 0.05) (Table 2). When the 7th day results were evaluated, the highest pH value was found in the GCK‐0.5 group (*p* < 0.05). The most acidic group was found to be IK‐0.5, and there was a significant difference (*p* < 0.05). Since the fermentation process is not pasteurized in kefir produced by the traditional method, the bacteria and yeasts in kefir that is ready for consumption continue their vitality. Accordingly, a decrease in the pH values of kefirs can be observed as the storage time increases (Farnworth 2005). In a study by Barukčić et al. (2017), the pH value of kefir groups varied between 4.58 and 4.65. In a study of kefir enriched with chickpea mucilage, pH decreased with storage time in control kefir, kefir with inulin, and kefir with chickpea mucilage (Ould Saadi et al. 2020). In a study by Irkin and Songun (İrkin and Songun 2022), the pH values of inulin‐added kefir and control group kefirs were compared, and it was reported that inulin addition did not affect the pH change in kefir samples, and the values ranged between 4.45 and 4.62. In a study conducted by Baniasadi et al. (2022), it was stated that the pH changes in kefir and yogurt samples during storage may be due to the different microbial populations of the milks used and the variability of their buffering capacities. It should be noted that monitoring pH in foods is important not only for flavor but also for safety, as excessive pH values can indicate spoilage or the growth of undesirable microorganisms (Travičić et al. 2023).

When the first day titration acidity results of kefir groups were analyzed, it was found that the IK‐0.5 group had the highest titration acidity value with 0.89 g/100 g lactic acid, while the GCK‐2 group had the lowest titration acidity value with 0.52 g/100 g lactic acid, which was statistically significant (*p* < 0.05) (Table 2). When the 7th day titration acidity results of kefir groups were analyzed, the highest titration acidity value was observed in the IK‐1 group with 0.83 g/100 g lactic acid. The lowest titration acidity was found in the GCK‐2 kefir group with 0.55 g/100 g lactic acid, and the differences in these results were statistically significant (*p* < 0.05). The titration acidity values determined in kefirs are consistent with the pH results. Since high yeast content limits the proliferation of lactic acid bacteria and acetic acid production, titratable acidity may not increase with increasing storage time. The reason for the significantly higher inulin acidity and lower pH values in the inulin group may be that yeast content could not be detected in the inulin‐added kefir group. Changes in acidification provide information about the growth patterns of bacteria in kefir samples. As the probiotic culture grows, it produces acid, which causes a decrease in pH (Chandrasekara and Shahidi 2011).

According to the results given in Table 2, the IK‐2 group had the highest dry matter content with 7.7% on the 1st day of storage, while the lowest dry matter content was found in the control kefir and the difference was statistically significant (*p* < 0.05). According to the study conducted by Kök‐Taş et al. (2013), the dry matter values of kefirs fermented with kefir grains and kefir culture were found to be 7.98% and 8.19% on Day 1, respectively. In a study conducted by Akan (2020), the total dry matter content ranges of kefir were found to be 10.41% (Day 6) and 11.11% (Day 0) during 7 days of storage. In the study conducted by Atalar (2019), total dry matter ranging between 9.32% and 9.43% and Öner et al. (2010) between 10.85% and 11.15% were determined according to storage days. In a study on kefir production from chickpea, rice, and almond milk, it was reported that the dry matter content was 6.94% (Ustaoğlu‐Gençgönül et al. 2024). The variability of these values is thought to be related to the characteristics of milk and the activity of starter cultures used in production. The level of water soluble solids is also related to the amount of sugar in kefir (Santos et al. 2016).

As can be seen in Table 3, the highest phenolic content among the kefir groups was observed in GCK‐2 with 341.12 mg GAE/100 mL according to the 1st day storage results. The lowest phenolic content was found in control kefir with 85.17 mg GAE/100 mL, and the difference was statistically significant (*p* < 0.05). In the study evaluating the total phenolic content of kefirs enriched with crumpled lentils and germinated crumpled lentils, the total phenolic content of kefir enriched with germinated crumpled lentils was found to be higher (Gunenc et al. 2017). In our study, the high phenolic content of germinated chickpea showed a positive effect on kefir, and the highest total phenolic content was observed in the GCK‐2 sample. Germination can cause oxidative stress in the plant, and therefore, the plant may produce more phenolics during germination because phenolics are involved in the plant defense system and provide antioxidant activity (Gunenc et al. 2017). In this regard, the results of the study were consistent with the release of greater amounts of phenolics as a defense mechanism during germination.

**TABLE 3:** TPC and antioxidant activity of kefir groups.

TPC (mg GAE/100 mL) (X̄±SD)			Antioxidant activity (DPPH)	
Samples	Day 1	Day 7	Day 1	Day 7
C	85.17 ± 4.45^g‐A^	105.79 ± 4.73^ı‐A^	1.16 ± 0.05^c‐A^	1.13 ± 0.05^ab‐A^
NGCK‐0.5	106.29 ± 2.92^e‐A^	140.99 ± 11.89^f‐A^	1.15 ± 0.03^c‐A^	1.11 ± 0.02^b‐A^
NGCK‐1	105.28 ± 1.37^e‐B^	190.77 ± 2.63^e‐A^	1.16 ± 0.03^c‐A^	1.11 ± 0.05^b‐A^
NGCK‐2	119.87 ± 2.98^d‐B^	263.68 ± 5.41^d‐A^	1.17 ± 0.01^c‐A^	1.12 ± 0.05^ab‐A^
IK‐0.5	110.81 ± 6.89^e‐A^	112.83 ± 2.94^hı‐A^	1.54 ± 0.07^a‐A^	1.12 ± 0.02^ab‐B^
IK‐1	94.72 ± 2.20^f‐B^	121.88 ± 3.41^gh‐A^	1.52 ± 0.09^a‐A^	1.12 ± 0.04^ab‐B^
IK‐2	103.27 ± 1.48^e‐A^	130.93 ± 1.93^fg‐A^	1.50 ± 0.07^a‐A^	1.12 ± 0.02^ab‐B^
GCK‐0.5	219.43 ± 3.68^c‐B^	284.80 ± 2.94^c‐A^	1.31 ± 0.04^b‐A^	1.12 ± 0.03^ab‐B^
GCK‐1	289.33 ± 4.07^b‐B^	358.72 ± 7.83^b‐A^	1.22 ± 0.04^bc‐A^	1.19 ± 0.02^ab‐A^
GCK‐2	341.12 ± 7.12^a‐B^	510.58 ± 9.11^a‐A^	1.49 ± 0.02^a‐A^	1.16 ± 0.04^ab‐B^

*Note:* Different lowercase letters in the same column indicate a significant difference between groups. (*p* < 0.05). Different capital letters on the same line indicate a significant difference between the groups (*p* < 0.05).

According to the results of the 1st day of storage, the highest total antioxidant activity values were determined in IK‐0,5, IK‐1, IK‐2, and GCK‐2 groups. There is a statistically significant difference between these kefir groups and other kefirs (*p* < 0.05) (Table 3). Similar to our findings, higher antioxidant activity was found in kefir samples supplemented with germinated lentils. The reason for this was reported to be that active antioxidant compounds may be released from dietary fiber during fermentation, leading to a higher antioxidant activity in kefir samples (Gunenc et al. 2017). A study has shown that kefir enriched with lentils has up to a 270% increase in antioxidant activity compared to kefir not enriched with lentils (Oliveira and Castro 2020). In another study, the antioxidant capacity of kefirs to which fruit juice was added decreased 72 h after fermentation. This may be due to the structure of phenolic compounds that can be affected by the activity of microbial enzymes, which can convert them into other molecules and affect the antioxidant activity of the beverage (Esatbeyoglu et al. 2023). Similarly, in our study, a decrease in the antioxidant activity of kefir groups was observed as the storage time increased. It is generally unexpected that DPPH antioxidant activity decreases while total phenolic content increases. This is because phenolic compounds generally have strong antioxidant properties, and increasing the amount of these compounds increases the capacity to react with the DPPH radical; that is, higher antioxidant activity is expected (Michalska et al. 2008). In this study, kefirs with low total phenolic content were observed to have high antioxidant activity (Table 3). In some cases, however, DPPH antioxidant activity may decrease or remain constant, even if the total amount of phenolics increases. The reasons for this situation may be as follows: *Structure of Phenolic Compounds*: Different phenolic compounds may have different antioxidant activity. For example, some phenolic compounds may interact more effectively with the DPPH radical, while others may interact less. If the structure of increased phenolic compounds consists of compounds that interact weakly with the DPPH radical, antioxidant activity may decrease even if the total amount of phenolics increases (Yamauchi et al. 2024). *Interaction of Phenolic Compounds with Other Compounds*: Phenolic compounds can lose their antioxidant activity by interacting with other compounds. Such interactions can occur especially in mixed matrices and may inhibit the reaction of phenolic compounds with the DPPH radical (Czubinski and Dwiecki 2017). *Complexity of the Matrix*: The matrix of the analyzed sample can affect the capacity of phenolic compounds to react with DPPH. For example, some substances may mask or inhibit the antioxidant activity of phenolic compounds. Therefore, while it is a possible scenario that DPPH antioxidant activity decreases as the total amount of phenolics increases, this may not always reflect the activity of phenolic compounds (Platzer et al. 2021). A study showed that the fermentation process of milk with traditional kefir increased its antioxidant activity by synthesizing phenolic compounds, but in commercial kefir, the antioxidant capacity was reduced by ABTS. Therefore, although the phenolic compounds produced by the starter culture seem to exert antioxidant action through their reducing power, they may have been ineffective in terms of the scavenging ability of oxidized compounds (Satir and Guzel‐Seydim 2015). In a study on kefir fermented from cow's milk, the total phenolic content of cow's milk was higher than that of kefir, but the antioxidant activity of cow's milk was lower than that of kefir, and total phenolic content and antioxidant activity were inversely correlated (Yirmibeşoğlu and Öztürk 2020). It is thought that this may be due to the metabolic activity of microorganisms in kefir grain.

When the kefir groups given in Table 4 were examined, it was found that IK‐0.5 had the highest Ca content with 154.1 mg/100 g on the 1st day of storage, while GCK‐2 group had the lowest Ca content with 106.2 mg/100 g and the difference between the groups was statistically significant (*p* < 0.05). When the P contents in kefir groups were evaluated, it was found that the control kefir (803.3 mg/L) and IK‐2 (787.1 mg/L) groups had the highest P contents on the 1st day of storage, while IK‐0.5 (194.5 mg/L), GCK‐0.5 (173.9) and GCK‐1 (194.1 mg/L) groups had the lowest P contents and the difference between the groups was statistically significant (*p* < 0.05). Although a significant number of reports presenting the mineral content of dairy products have been published so far, there is limited research on the mineral composition of kefir products. It is known that the fermentation process can alter the distribution of essential elements in kefir (de Oliveira et al. 2019). Ozcan (2010) investigated the mineral content of industrial kefir samples collected from markets in Turkey. The researcher focused on Ca and P minerals, which are major minerals in milk, and the Ca and P contents of plain kefir were found to be 1.28 and 8.71 g/kg, respectively. According to the results obtained in our study, the highest P mineral values were obtained in the control group. According to this result, it was determined that P mineral was negatively affected by different treatments applied to kefir. When the Ca mineral results were analyzed, it was observed that the amount of Ca mineral decreased as the treatment rates increased. It was evaluated that this was due to the fact that Ca mineral binds more and its absorption decreases as the amount of treated components increases.

**TABLE 4:** Mineral contents of kefir groups.

Ca (X̄±SD) (mg/100 g)			P (X̄±SD) (mg/L)	
Samples	Day 1	Day 7	Day 1	Day 7
C	149.8 ± 4.09^ab‐A^	150.2 ± 2.33^a‐A^	803.3 ± 19.05^a‐A^	825.5 ± 22.21^a‐A^
NGCK‐0.5	150.1 ± 0.91^ab‐A^	148.2 ± 1.60^a‐A^	426.2 ± 11.55^d‐A^	416.5 ± 10.53^d‐B^
NGCK‐1	139.1 ± 4.45^c‐A^	135.5 ± 1.08^b‐A^	386.6 ± 10.84^e‐A^	370.4 ± 22.58^e‐A^
NGCK‐2	141.7 ± 1.90^c‐A^	140.1 ± 2.17^b‐A^	525.4 ± 10.18^c‐B^	545.2 ± 5.99^c‐A^
IK‐0.5	154.1 ± 3.51^a‐A^	153.4 ± 3.08^a‐A^	194.5 ± 5.16^f‐A^	205.3 ± 7.14^f‐A^
IK‐1	150.2 ± 4.12^ab‐A^	148.6 ± 6.15^a‐A^	356.3 ± 7.06^e‐B^	375.2 ± 5.40^e‐A^
IK‐2	145.8 ± 1.34^bc‐A^	140.3 ± 1.77^b‐A^	787.1 ± 6.06^a‐A^	602.9 ± 4.55^b‐B^
GCK‐0.5	125.2 ± 3.42^d‐A^	122.5 ± 0.87^c‐A^	173.9 ± 2.98^f‐A^	165.3 ± 3.59^g‐A^
GCK‐1	121.3 ± 8.51^d‐A^	122.6 ± 6.39^c‐A^	194.1 ± 51.41^f‐A^	222.2 ± 9.15^f‐A^
GCK‐2	106.2 ± 2.76^e‐A^	105.5 ± 5.98^d‐A^	709.2 ± 20.73^b‐A^	608.6 ± 17.93^b‐B^

*Note:* Different lowercase letters in the same column indicate a significant difference between groups. (*p* < 0.05). Different capital letters on the same line indicate a significant difference between the groups (*p* < 0.05).

On the 1st day of storage when kefir samples were evaluated, the IK‐1 group had the highest LAB count with 8.61 log cfu/mL. The lowest LAB count was found in the control kefir group with 7.69 log cfu/mL and the results were statistically significant (*p* < 0.05). On the 7th day of storage, the highest LAB count was found in IK‐1 and IK‐2 groups and was statistically significant (*p* < 0.05), but there was no significant difference between IK‐1 and IK‐2 groups (*p* > 0.05) (Table 5). Which species predominates in kefir grains varies depending on the geographical origin of the grains, substrate and fermentation conditions (Lynch et al. 2021). In yogurt production, legumes, including chickpea flour, facilitated the development of probiotic bacteria by enhancing the growth of lactobacilli (Zare et al. 2012). In a study on the fermentation of chickpea milk, the change in the number of microorganisms increased significantly after fermentation (*p* < 0.05).

**TABLE 5:** Microbial findings of kefir groups.

LAB			TMAB		Yeast	
(log kob/mL) (X̄±SD)			(log kob/mL) (X̄±SD)		(log kob/mL) (X̄±SD)	
Samples	Day 1	Day 7	Day 1	Day 7	Day 1	Day 7
C	7.69 ± 0.20^f‐A^	7.75 ± 0.16^d‐A^	7.84 ± 0.69^cd‐A^	7.77 ± 0.35^d‐A^	ND	ND
NGCK‐0.5	8.27 ± 0.13^bcde‐A^	8.32 ± 0.10^abc‐A^	8.23 ± 0.22^bcd‐A^	8.18 ± 0.48^bcd‐A^	2.07 ± 0.99^bc‐A^	2.30 ± 1.18^b‐A^
NGCK‐1	8.49 ± 0.05^abc‐A^	8.35 ± 0.12^abc‐A^	8.54 ± 0.26^ab‐A^	8.60 ± 0.33^ab‐A^	1.30 ± 0.33^c‐A^	2.17 ± 0.87^b‐A^
NGCK‐2	8.30 ± 0.25^abcd‐A^	8.32 ± 0.10^abc‐A^	8.34 ± 0.40^abcd‐A^	8.45 ± 0.22^ab‐A^	3.11 ± 0.87^ab‐A^	2.17 ± 0.57^b‐A^
IK‐0.5	7.95 ± 0.22^def‐A^	8.01 ± 0.15^cd‐A^	8.11 ± 0.28^bcd‐A^	8.01 ± 0.13^cd‐A^	ND	ND
IK‐1	8.61 ± 0.12^a‐A^	8.62 ± 0.19^a‐A^	8.41 ± 0.11^abc‐A^	8.21 ± 0.14^bcd‐A^	ND	ND
IK‐2	8.53 ± 0.21^ab‐A^	8.55 ± 0.37^a‐A^	8.39 ± 0.12^abc‐A^	8.22 ± 0.11^bcd‐A^	ND	ND
GCK‐0.5	7.99 ± 0.13^def‐A^	8.05 ± 0.14^bcd‐A^	7.77 ± 0.15^d‐A^	7.89 ± 0.18^d‐A^	4.36 ± 0.87^a‐A^	4.19 ± 0.87^a‐A^
GCK‐1	8.36 ± 0.18^abc‐A^	8.42 ± 0.27^ab‐A^	8.84 ± 0.18^a‐A^	8.75 ± 0.14^a‐A^	4.54 ± 0.57^a‐A^	4.59 ± 0.87^a‐A^
GCK‐2	8.17 ± 0.13^de‐A^	8.29 ± 0.18^abc‐A^	8.25 ± 0.12^bcd‐A^	8.38 ± 0.07^abc‐A^	4.24 ± 0.66^a‐A^	4.55 ± 0.66^a‐A^

*Note:* Different lowercase letters in the same column indicate a significant difference between groups. (*p* < 0.05). Different capital letters on the same line indicate a significant difference between the groups (*p* < 0.05).

Abbreviation: ND, non detected.

The results showed that LAB had good growth in chickpea milk and accumulated rapidly after 24 h of fermentation, reaching 8.87 log CFU/mL (Zhang et al. 2022).

When the total mesophilic aerobic bacteria count was analyzed, the highest TMAB was found in the GCK‐1 group with 8.84 log cfu/mL, and the lowest TMAB was found in the GCK‐0.5 group with 7.77 log CFU/mL. According to the 7th day results, the number of TMABs was similar in the groups to which inulin was added, while the highest differentiation was detected in GCK samples (Table 5). In a study conducted by Kezer (2022), the total mesophilic aerobic bacteria counts of commercially produced fruity, plain, and lactose‐free kefir samples sold in Kırşehir and kefirs produced from kefir grains by traditional methods were examined and found to be 7.08, 7.08, 7.21, and 9.30 log CFU/mL, respectively. In a study investigating kefir enriched with chickpea mucilage, all kefir samples showed a significant increase in total viable count on Day 7 compared to Day 1. After Day 7, the number remained constant until Day 21 and then decreased (Ould Saadi et al. 2020). Taş et al. (2014) found that the number of TMABs decreased during storage in kefir samples with plum addition. In another study conducted by Karabiyikli and Daștan (2016), the total mesophilic aerobic bacteria count was examined to determine the microbiological profiles of kefir and kefir grain samples, and it was found to be between 5.74 and 8.50 log CFU/mL. In this study, the number of TMABs decreased during storage except for the NGCK‐1, NGCK‐2, GCK‐0.5, and GCK‐2 groups. The addition of chickpeas can provide positive effects on the microbial profile of kefir. Chickpeas contain nutrients and fiber that help probiotics grow, which can support bacterial proliferation.

When kefir groups were analyzed, yeast was not detected in control kefir and inulin added kefir group. The highest yeast counts were observed in the GCK groups on Day 1 and 7 and were found to be significantly higher than the control group (*p* < 0.05) (Table 5). This may be explained by the fact that germinated chickpeas may provide a suitable food source for yeasts and promote their reproduction. In particular, free amino acids, sugars, and other nutrients released during the germination process may have supported the growth of yeasts (Boyaci Gunduz and Erten 2022). Inulin has also been shown in some studies to limit yeast growth in certain environments or to suppress yeast growth, giving bacteria a greater advantage (Kaya 2015).

**FIGURE 4:**
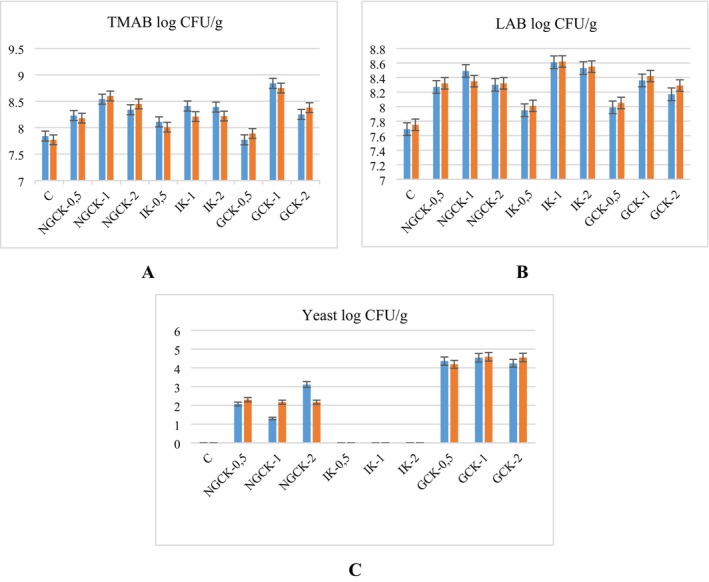
(A) Total mesophilic aerobic bacteria (TMAB), (B) lactic acid bacteria (LAB), (C) yeast content of kefir group on Day 1 and 7, 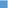 Day 1, 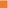 Day 7.

The number of LAB was investigated during 28 days of storage at 4°C of a fermented kefir beverage in Brazil. During this period, the LAB group numbers remained constant until the end of the storage period (Leite et al. 2013). Grønnevik et al. (2011) found that the number of LAB in kefir samples decreased during the first 4 weeks of storage, whereas yeast levels increased during the storage period. Montanuci et al. (2012) reported that yeast, acetic acid bacteria and *Leuconostoc* numbers increased at the end of storage, while LAB and *Lactococcus* numbers decreased compared to the beginning of storage. In this study, it was determined that kefir groups enriched with chickpea and germinated chickpea had higher LAB counts than the control kefir group, and it was found that this difference was statistically significant (*p* < 0.05) (Figure 4). This is thought to indicate that germinated chickpea and inulin have similar prebiotic activity.

According to the sensory analysis results of the kefir groups on the 1st and 7th days of storage at 4°C ± 1°C, the highest scores in appearance, odor, taste and texture were obtained from the control kefir and the difference was statistically significant (*p* < 0.05). IK group was significantly higher than the control kefir in all properties (*p* < 0.05). There was a significant decrease in appearance, taste, odor and texture scores of the GCK group as the chickpea inclusion rate increased (*p* < 0.05) (Table 6).

**TABLE 6:** Sensory analysis results of kefir groups.

Kefir groups	Storage day	Appearance	Smell	Taste	Texture	Overall score
**C**	1	9.9 ± 0.30^a^	10.0 ± 0^a^	10.0 ± 0^a^	9.8 ± 0.6^a^	9.92 ± 0.34^a^
C	7	9.8 ± 0.4^a^	9.8 ± 0.41^a^	9.8 ± 0.40^a^	9.6 ± 0.66^a^	9.75 ± 0.48
GCK‐0.5	1	7.1 ± 1.70^bcd^	7.9 ± 1.86^b^	4.6 ± 3.07^de^	5.8 ± 1.46^cde^	6.35 ± 2.46^d^
GCK‐0.5	7	6.2 ± 1.24^bcd^	6.0 ± 1.73^b^	4.4 ± 2.57^de^	5.5 ± 1.11^cde^	5.52 ± 1.89
GCK‐1	1	6.1 ± 1.04^d^	4.1 ± 1.51^c^	3.4 ± 2.20^e^	4.2 ± 1.93^ef^	4.45 ± 1.99^ef^
GCK‐1	7	5.2 ± 1.16^d^	3.4 ± 0.91^c^	3.4 ± 1.42^e^	3.8 ± 1.32^ef^	3.95 ± 1.43
GCK‐2	1	4.4 ± 1.42^e^	3.8 ± 0.97^c^	3.3 ± 2.10^e^	2.8 ± 1.53^f^	3.57 ± 1.67^f^
GCK‐2	7	4.0 ± 1.34^e^	3.6 ± 0.91^c^	3.4 ± 1.20^e^	3.1 ± 1.44^f^	3.52 ± 1.28
IK‐0.5	1	7.8 ± 1.46^bc^	8.0 ± 1.84^b^	8.1 ± 1.64^b^	7.0 ± 2.96^bcd^	7.72 ± 2.10^bc^
IK‐0.5	7	7.1 ± 1.30^bc^	7.3 ± 1.41^b^	8.3 ± 1.10^b^	6.7 ± 2.79^bcd^	7.35 ± 1.87
IK‐1	1	8.5 ± 1.74^b^	7.1 ± 1.7^b^	8.9 ± 0.94^ab^	8.2 ± 1.16^ab^	8.17 ± 1.57^b^
IK‐1	7	7.8 ± 1.53^b^	6.5 ± 1.56^b^	8.7 ± 0.78^ab^	7.6 ± 1.01^ab^	7.65 ± 1.49
IK‐2	1	7.3 ± 1.26^bcd^	7.5 ± 2.06^b^	7.3 ± 1.67^bc^	7.6 ± 2.45^bc^	7.42 ± 1.92^bc^
**IK‐2**	7	6.3 ± 1.26^bcd^	7.0 ± 1.26^b^	7.2 ± 1.16^bc^	7.1 ± 2.11^bc^	6.9 ± 1.54
NGCK‐0.5	1	8.4 ± 0.91^b^	7.5 ± 1.36^b^	5.6 ± 1.95^cd^	7 ± 1.61^bcd^	7.12 ± 1.81^cd^
NGCK‐0.5	7	6.8 ± 1.46^b^	6.6 ± 1.11^b^	5.0 ± 1.54^cd^	6.3 ± 1.18^bcd^	6.17 ± 1.51
NGCK‐1	1	4.5 ± 2.20^e^	5.0 ± 2.14^c^	3.4 ± 1.90^e^	2.5 ± 1.20^f^	3.85 ± 2.13^f^
NGCK‐1	7	3.8 ± 1.53^e^	3.8 ± 1.24^c^	3.7 ± 1.26^e^	3.1 ± 1.51^f^	3.6 ± 1.42
NGCK‐2	1	6.7 ± 1.73^cd^	5.4 ± 2.10^c^	3.0 ± 1.48^e^	5.6 ± 1.95^de^	5.17 ± 2.27^e^
NGCK‐2	7	5.4 ± 1.42^cd^	3.8 ± 1.40^c^	3.2 ± 1.24^e^	3.8 ± 1.88^de^	4.05 ± 1.71

*Note:* Different lowercase letters in the same column indicate a significant difference between groups. (*p* < 0.05).

**FIGURE 5:**
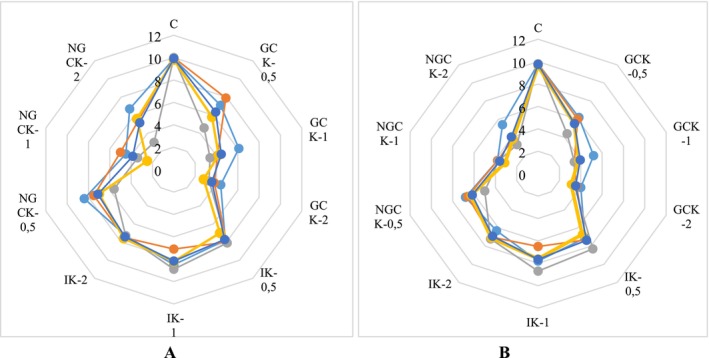
Sensory analysis results of kefir groups. (A) Day 1 sensory analysis results; (B) Day 2 sensory analysis results; 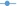: appearaence; 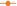: smell, 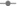: taste, 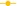: texture, 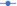: overall score).

Descriptive results of sensory analyses are often expressed by a spider web diagram. Figure 5 shows the results of the sensory analysis of kefir groups.

Glibowski and Zielińska (2015) conducted a study examining the sensory analysis results of kefirs with and without inulin and found no differences between the groups in terms of texture and aroma. Additionally, no significant differences in taste were found between fat‐free kefirs with or without inulin. In this study, the inulin‐added group (IK) was found to be significantly higher in all attributes compared to the control kefir (*p* < 0.05) (Figure 5).

The fermentation of lactic acid bacteria significantly reduces the chickpea aroma in kefir. LAB fermentation may provide a characteristic aroma to chickpea‐supplemented kefir by producing active reductases and unique aromatic compounds (Blagden and Gilliland 2005). Moreover, LAB fermentation can convert certain undesirable aldehydes into corresponding alcohols and acids with fruity and sweet notes (Tangyu et al. 2021). In this study, as the chickpea incorporation ratio increased in the GCK group, there was a significant decrease in appearance, taste, aroma, and texture scores (*p* < 0.05). Yeasts contribute to the flavor and mouthfeel of kefir by altering the pH, secreting ethanol, and producing CO_2_ (Farnworth 2005). However, the anticipated effects commonly reported in the literature were not fully observed, which may be attributed to the relatively high incorporation ratio of germinated chickpeas used in the formulation. This study enhanced the nutritional value and quality of kefir produced with the addition of germinated chickpeas by increasing the functional value of low‐value chickpeas.

The aim of this study was to develop a novel functional product that would be acceptable to consumers due to its enhanced nutritional properties. However, the sensory evaluation results indicated that the desired outcomes were not fully achieved. Specifically, as the incorporation levels of non‐germinated chickpea (NGCK) and germinated chickpea (GCK) into kefir increased, a statistically significant decline in sensory attributes, namely appearance, aroma, taste, and texture, was observed.

These findings align with previous studies reporting that although legume‐based enrichment improves nutritional and functional qualities, it can negatively influence the sensory acceptance of fermented dairy products (Wajs et al. 2023; Bulut et al. 2022). The beany or earthy flavors imparted by legumes, particularly at higher concentrations, have been identified as limiting factors in consumer acceptance (Vurro et al. 2024). Furthermore, the presence of fibrous or particulate matter from chickpeas may alter the mouthfeel and texture, contributing to reduced sensory scores (Elbahnasi et al. 2021).

While germination is known to improve flavor and reduce off‐notes by lowering antinutritional factors (Badjona et al. 2023), it appears that the chickpea concentration used in this study may have surpassed the sensory tolerance threshold for fermented dairy matrices. Similar observations were made by Atudorei and Codină (2020) who noted that incorporating germinated legume flours beyond certain ratios led to decreased overall acceptability in yogurt‐like products.

These outcomes underscore the need for optimizing incorporation levels and possibly combining chickpea supplementation with flavor‐masking agents or complementary ingredients to balance functional benefits with sensory appeal in future formulations.

**FIGURE 6:**
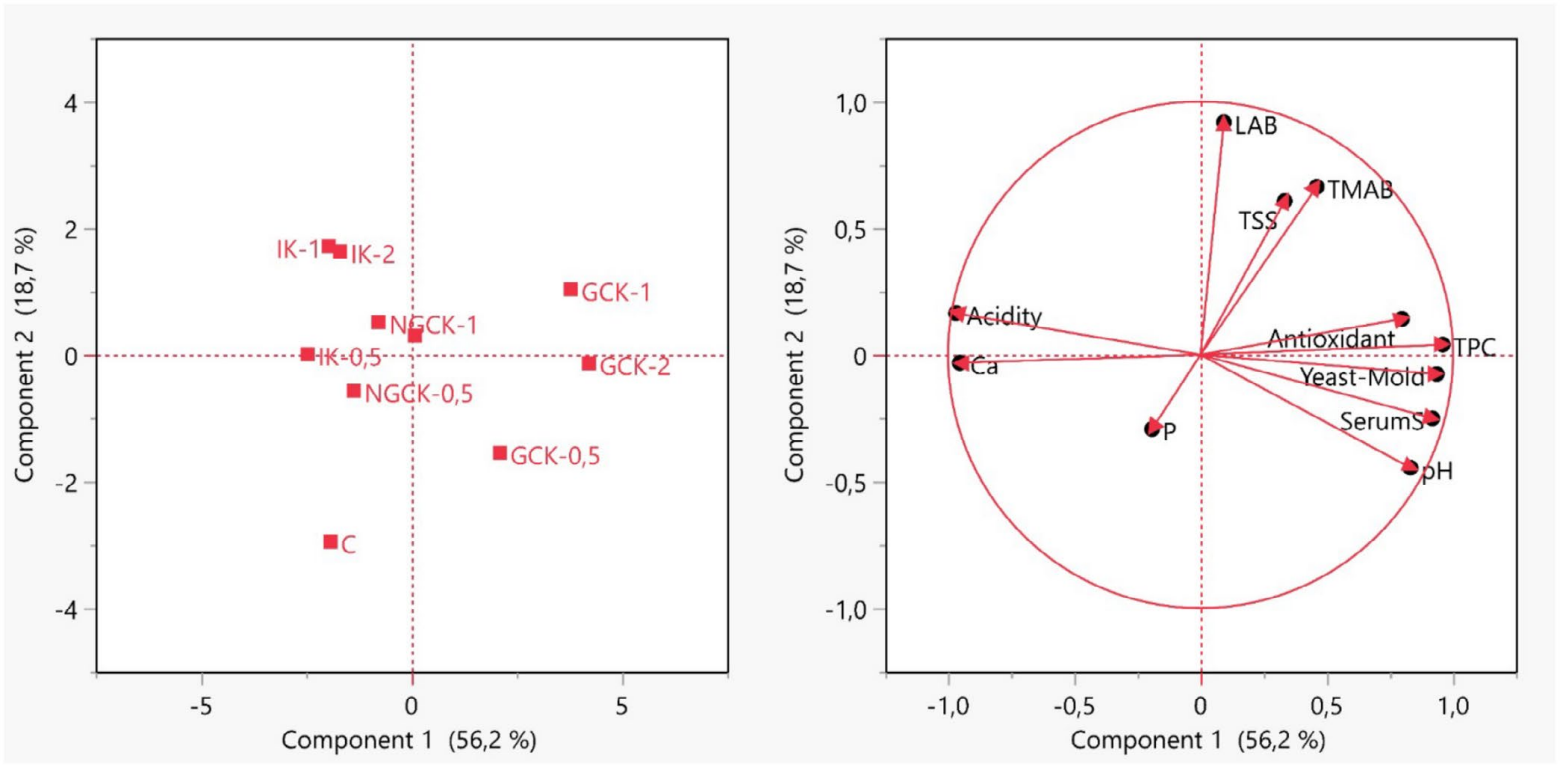
Evaluation of physicochemical, microbiological, and sensory analyses of kefir groups by PCA.

In Figure 6, the projections of the variables used in the study onto the two factors with the highest variance are shown. After PCA analysis, it was determined that the two principal components explained 74.9% of the total variance. The variances of these two factors were calculated as 56.2% and 18.7%, respectively. Twelve different analyses (pH, titration acidity, water‐soluble dry matter, serum separation, calcium content, phosphorus content, total phenolic content, antioxidant activity, total mesophilic aerobic bacteria count, lactic acid bacteria count, yeast count, and sensory analyses) were applied to the 10 groups and included in the PCA.

When the PCA analysis results of kefirs produced with different treatment methods were examined, it was determined that the GCK‐1 group is located in the upper right area of the plane and is the group positively affected by the two principal factors. It was determined that the separation of the GCK group from the other groups was due to the high values revealed in analyses such as lactic acid bacteria count, total phenolic content, total mesophilic aerobic bacteria count, yeast count, and antioxidant activity. It was determined that the negative effects on all groups stemmed from the sensory analysis, as well as the calcium (Ca) and phosphorus (P) results. Based on this conclusion, it was assessed that the different treatment methods had a negative impact on the sensory and mineral outcomes of the kefir. According to the PCA results, it was determined that the GCK‐1 group had the most optimal results among the kefirs obtained with different treatment methods.

## 4 Conclusion

This study aimed to enhance the nutritional composition of chickpeas through germination and to investigate the physicochemical, microbiological, and sensory properties of kefir enriched with germinated chickpeas, a traditional fermented beverage. As a result of the study, the GC‐8 group, with a germination duration of 72 h at 30°C and 12 h of soaking, showed an increase in protein and total phenolic content and was identified as the most optimal group to be added to the kefir.

The produced kefirs were divided into 10 groups: control, inulin, germinated chickpea, and non‐germinated chickpea added groups. In the germinated chickpea added kefir samples, higher total phenolic content and antioxidant activity were found after fermentation, but lower mineral levels were observed. The superior microbiological properties of the germinated chickpea added kefirs have made them stand out compared to the other kefir groups. In the study, it was expected that the inulin‐added kefir group would exhibit prebiotic activity, and it was found that the LAB count was high. The fact that the LAB count in the germinated chickpea added kefir group was at a competitive level with the inulin‐added kefir group, along with the observation of yeasts only in the chickpea‐added groups, was considered a positive effect.

It can be concluded that enriching kefir with germinated chickpeas affects the nutritional content and composition of kefir in various ways in this study. These effects have varied depending on the chickpea incorporation ratio and the method used. However, further research is needed to explain the underlying mechanisms driving these changes. The changes observed in this study highlight the importance of considering these variables when optimizing the processing methods of chickpeas to achieve the best nutritional profile for kefir. Therefore, it contributes to the development of processing techniques that preserve or enhance the nutritional value of kefir enriched with germinated chickpeas and promote its use as a source of health‐promoting compounds in human nutrition.

## Author Contributions

**Ayşe Nur Kahve:** conceptualization (equal), data curation (equal), formal analysis (equal), funding acquisition (equal), investigation (equal), methodology (equal), resources (equal), software (equal), visualization (equal), writing – original draft (equal), writing – review and editing (equal). **Ebru Bayrak:** conceptualization (equal), data curation (equal), funding acquisition (equal), methodology (equal), project administration (equal), supervision (equal), validation (equal), writing – review and editing (equal).

## Ethics Statement

The authors have nothing to report.

## Conflicts of Interest

The authors declare no conflicts of interest.

## Data Availability

The datasets generated during and/or analyzed during the current study are available from the corresponding author on a reasonable request.
